# Mitoregulin Contributes to Creatine Shuttling and Cardiolipin Protection in Mice Muscle

**DOI:** 10.3390/ijms24087589

**Published:** 2023-04-20

**Authors:** Olga A. Averina, Oleg A. Permyakov, Mariia A. Emelianova, Olga O. Grigoryeva, Maxim L. Lovat, Anna E. Egorova, Andrei V. Grinchenko, Vadim V. Kumeiko, Maria V. Marey, Vasily N. Manskikh, Olga A. Dontsova, Mikhail Yu. Vysokikh, Petr V. Sergiev

**Affiliations:** 1 Institute of Functional Genomics, Lomonosov Moscow State University, 119992 Moscow, Russia; 2 Belozersky Institute of Physico-Chemical Biology, Lomonosov Moscow State University, 119992 Moscow, Russia; 3 Center for Life Sciences, Skolkovo Institute of Science and Technology, 143025 Moscow, Russia; 4 Institute of Mitoengineering MSU, 119992 Moscow, Russia; 5 Institute of Life Sciences and Biomedicine, Far Eastern Federal University, 690922 Vladivostok, Russia; 6 A.V. Zhirmunsky National Scientific Center of Marine Biology, 690041 Vladivostok, Russia; 7 Research Center for Obstetrics, Gynecology and Perinatology, 117198 Moscow, Russia; 8 Department of Chemistry, Lomonosov Moscow State University, 119991 Moscow, Russia; 9 Shemyakin-Ovchinnikov Institute of Bioorganic Chemistry, Russian Academy of Sciences, 119992 Moscow, Russia

**Keywords:** short open reading frame, small peptide, oxidative phosphorylation, mitochondria, metabolism, cardiolipin, creatine kinase

## Abstract

Small peptides compose a large share of the mitochondrial proteome. Mitoregulin (Mtln) is a mitochondrial peptide known to contribute to the respiratory complex I functioning and other processes in mitochondria. In our previous studies, we demonstrated that *Mtln* knockout mice develop obesity and accumulate triglycerides and other oxidation substrates in serum, concomitant with an exhaustion of tricarboxylic acids cycle intermediates. Here we examined the functional role of Mtln in skeletal muscles, one of the major energy consuming tissues. We observed reduced muscle strength for Mtln knockout mice. Decrease of the mitochondrial cardiolipin and concomitant increase in monolysocardiolipin concentration upon *Mtln* inactivation is likely to be a consequence of imbalance between oxidative damage and remodeling of cardiolipin. It is accompanied by the mitochondrial creatine kinase octamer dissociation and suboptimal respiratory chain performance in *Mtln* knockout mice.

## 1. Introduction

Small peptides compose a large group of molecules whose contribution to the functioning of live organisms started to emerge only recently (see [1,2,3,4,5] for review). Particularly, a diverse set of peptides are used by vertebrates to fine-tune muscle performance [6,7,8]. One of those peptides identified recently is a mitochondrial peptide Mtln [9,10,11]. The peptide Mtln was previously demonstrated to interact with CYB5R3 [9], a trifunctional protein involved in β-oxidation [11,12] and ATP synthase [12] as well as to assist respiratory complexes association into supercomplexes [10].

Skeletal muscles are known to consume a large share of energy that is produced by oxidative phosphorylation and glycolysis. Accordingly, muscle phenotypes are frequently observed for genetic diseases whose molecular mechanisms involve mitochondrial malfunction [13,14]. Among the phenotypes observed in *Mtln* knockout mice are lower performance on a treadmill or rotarod [11,15], but not without some controversies [10], reduction in the grip strength [15,16], smaller myofibrils [15,16], again with some doubts [11], and a number of respiratory phenotypes measured ex vivo on isolated muscle tissue samples [10,11] and mitochondria [11,15] as well as on the *Mtln* knockout cell cultures [9,12]. The conditions which lead to the manifestation of respiratory defects vary from study to study. While respiration on glutamate/malate [9] or glucose/glutamate/pyruvate [15] was found to be decreased for mitochondria of *Mtln* knockout cells, other studies performed on mice tissues detected respiratory defects only on the fatty acid substrate palmitoyl carnitine [10,11,12], in some cases only after animal fasting [10], but not on other substrates. Knockout mice body mass measurement also gained controversial results from no dependence on Mtln functionality [11,12,15], to decreased [16] or increased weight of knockout mice [17]. Triglycerides accumulation upon *Mtln* inactivation was reported for fibroblasts and myeloma cells [9] as well as for adipocytes [12] and knockout mice [17], although not in all studies [11].

To gain further insight into the controversial issue of Mtln function at the level of an organism, we created a *Mtln* knockout mice line [17] carrying 82 nucleotide deletions encompassing the *Mtln* promoter region, which we refer to as Δ*Mtln-1*. In addition, to independently confirm major results, we used another *Mtln* knockout line obtained in our laboratory, which carries an 8 nt. deletion eliminating the start codon of this gene. This line is further referenced as Δ*Mtln-2*. Both lines were backcrossed three times to the C57Bl/6J line. Heterozygous carriers of the inactivating alleles were mated and the obtained homozygous progeny were used to establish Δ*Mtln-1* (Δ82/Δ82) and Δ*Mtln-2* (Δ8/Δ8) knockout and wild type control lines for all further experiments. The potential influence of the genetic background beyond the *Mtln* gene was minimized, although not completely excluded, as the control wild type mice line used originated from the littermates of knockout mice. In this work, we present our study of Mtln influence on muscle physiology.

## 2. Results

### 2.1. Muscle Strength of *Δ*Mtln Mice

**Figure 1:**
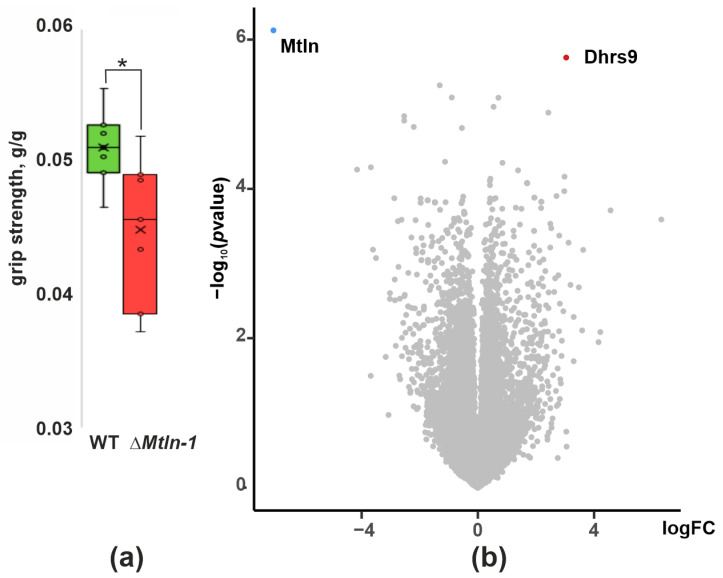
Influence of *Mtln* inactivation on integrative characteristics of muscle. (**a**) Forelimb grip strength of the wild type (green bar, *n* = 10) and Δ*Mtln-1* (red bar, *n* = 13) male mice after 24 h food deprivation; interquartile ranges are shown as solid bars, while the all-data range is shown by thin lines. The horizontal line corresponds to the median, while the cross to the average. The significance level calculated using the Student’s *t*-test is shown; If a *p*-value is less than 0.05, it is flagged with one star (*) (**b**) Differential gene expression in the soleus muscle of the wild type and Δ*Mtln-1* knockout mice. Volcano plot of differentially expressed genes. The x-axis corresponds to the log-scale fold change of expression, Δ*Mtln-1* relative to the wild type, while the y-axis corresponds to *p*-value.

Several lines of evidence suggest that Mtln plays a significant role in formation and function of muscle tissue [10,11,15,16]. We addressed this issue with our Δ*Mtln-1* knockout mice model we described earlier [17]. To evaluate muscle performance of the Δ*Mtln-1* knockout mice, we applied the grip strength test after 24 h food deprivation (Figure 1a). As a result, we observed a decrease in muscle performance caused by *Mtln* gene inactivation in agreement with previously published findings [15,16]. To pursue this further, the performance of muscle was assessed by the observation of the electro-stimulated contraction of tibialis anterior, soleus and gastrocnemius ex vivo for the wild type and Δ*Mtln-1* knockout mice, which were deprived of food for 24 h (Supplementary Figure S1). However, no statistically significant reduction in electro-stimulated muscle strength was demonstrated (Supplementary Figure S1).

### 2.2. Influence of Mtln Gene Inactivation on Muscle Transcriptome

To analyze whether *Mtln* gene inactivation resulted in a differential gene expression in the muscle, we compared the soleus muscle transcriptome of the Δ*Mtln-1* mice line with that of the wild type control (Figure 1b, Supplementary Tables S1–S3) and revealed only minor changes. The most significantly downregulated gene is, in agreement with expectations, *Mtln*. Deletion of 82 nucleotides, which was generated in the Δ*Mtln-1* line, is likely to encompass the promoter region thus inhibiting transcription of the gene. Apart from *Mtln*, only *Dhrs9*, a desaturase involved in biosynthesis of several lipid soluble hormones, demonstrated a significant expression change (upregulation) in the Δ*Mtln-1* mice line. While we have not observed statistically significant differences in the expression of individual genes upon *Mtln* inactivation, we set up to analyze coordinate changes in expression of gene sets. To this end we used GSEA [18] molecular signature analysis and the gene ontology (GO) molecular function database [19,20]. As a result, we obtained a number of up- (Supplementary Table S2) and down-regulated gene sets (Supplementary Table S3). Among up-regulated sets of genes were several related to the immune system, e.g., MHC protein complex binding and immune receptor activity, as well as several sets related to fatty acid hydroxylation. The most significant down-regulated gene set was a structural constituent of the ribosome, including the mitochondrial one. The limited influence of *Mtln* inactivation on gene expression prompted us to seek a biochemical difference in muscles rather than gene expression perturbations.

### 2.3. Histopathological Analysis of *Δ*Mtln Mice Muscles

Muscles are divergent in their preferences for particular sources of energy. As both oxidative (soleus) and glycolytic (tibialis anterior) muscles were shown to contain the Mtln peptide (Supplementary Figure S2), we set up to analyze both muscles further. Histological analysis (Supplementary Figure S3) of soleus (Supplementary Figure S3e,h) and tibialis anterior (Supplementary Figure S3f,i) muscle from the wild type and Δ*Mtln-1* knockout mice revealed no difference in the diameter of myofibrils (Supplementary Figure S3j). The myofibers with a nuclear chain in the center were counted as a proxy for muscle remodeling (Supplementary Figure S3k). While the difference between the wild type and Δ*Mtln-1* knockout mice in regard to the proportion of myofibers with a nuclear chain in the center did not reach statistical significance, we observed a tendency towards increase in this parameter for knockout mice. To assess possible mitochondrial damage exacerbated by the *Mtln* knockout, we used Gomori trichrome stain on the soleus (Supplementary Figure S3l) and tibialis anterior muscle slices (Supplementary Figure S3m). While no significant damage of the muscle mitochondria was found in the Δ*Mtln-1* knockout mice using this approach, we could not rule out that more subtle differences might have been found using electron microscopy in the future research.

### 2.4. Respiration of Muscle Mitochondria from *Δ*Mtln Mice

**Figure 2:**
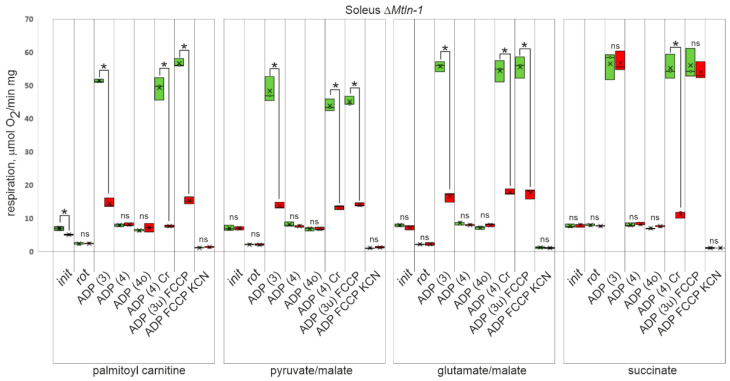
Influence of *Mtln* gene inactivation on respiration of muscle mitochondria. Oxygen consumption rate (OCR) of soleus muscle mitochondria extracted from the wild type (green bars, *n* = 3) and Δ*Mtln-1* (red bars, *n* = 3) male mice. The groups of bars correspond to the respiration on palmitoyl carnitine (CI + CII + ETF activity), pyruvate and malate (CI activity), glutamate and malate (CI activity) and succinate (CII activity) as marked below the graphs. The experimental points measured are substrates alone (init), substrates with rotenone (rot), substrates and ADP (ADP (3)), substrates after exhaustion of ADP (ADP (4)), substrates after exhaustion of ADP after addition of oligomycin (ADP (4o)), substrates after exhaustion of ADP (ADP (4)) following addition of creatine, substrates after exhaustion of ADP after addition of oligomycin and FCCP uncoupler (ADP (3u) FCCP), residual respiration after inhibition of uncoupled respiration by potassium cyanide. Interquartile ranges are shown as solid bars, while the all-data range is shown by thin lines. The horizontal line corresponds to the median, while the cross is the average. The significance level calculated using the Student’s *t*-test is shown. If a *p*-value is less than 0.05, it is flagged with one star (*).

Our laboratory [9] and other groups [10,11,12,15] have demonstrated previously, that *Mtln* gene knockout in cell lines and mice affects mitochondrial respiration, however, details on which respiration substrate utilization is specifically affected by the lack of Mtln are inconsistent. To address this issue, we isolated mitochondria from the oxidative (soleus) and glycolytic (tibialis anterior) muscles from the wild type, Δ*Mtln-1*, and Δ*Mtln-2* knockout mice, and measured their respiration rate using a fatty acid derivative palmitoyl carnitine (Figure 2, Supplementary Figure S4, 1st group of bars), or specific Complex I substrates pyruvate and malate (Figure 2, Supplementary Figure S4, 2nd group of bars), or glutamate and malate (Figure 2, Supplementary Figure S4, 3rd group of bars). The oxygen consumption rate on Complex II substrate succinate (Figure 2, Supplementary Figure S4, 4th group of bars) was used to assess respiration independent on Complex I activity. The following conditions were used to assess respiration efficiency on each of the above mentioned substrates: (i) oxidative substrate alone; (ii) oxidative substrate and Complex I inhibitor rotenone; (iii) oxidative substrate and excess of ADP, i.e., state 3 respiration; (iv) oxidative substrate after exhaustion of added ADP, i.e., state 4 respiration; (v) oxidative substrate after exhaustion of added ADP and ATP synthase inhibition with oligomycin, i.e., state 4o respiration; (vi) oxidative substrate after exhaustion of added ADP and addition of the excess of creatine, i.e., when ADP regeneration depends on creatine kinase coupling; (vii) oxidative substrate after exhaustion of added ADP and ATP synthase inhibition with oligomycin and with FCCP uncoupler addition, i.e., state 3u; (viii) oxidative substrate after exhaustion of added ADP and ATP synthase inhibition with oligomycin and with FCCP uncoupler addition and inhibition of Complex IV with cyanide, i.e., residual oxygen consumption. The results related to the soleus muscle mitochondria of the Δ*Mtln-1* mice are presented in Figure 2; data for the tibialis anterior muscle mitochondria of the Δ*Mtln-1* mice are presented in the Supplementary Figure S4a, while those for the soleus and tibialis anterior muscle mitochondria of the Δ*Mtln-2* mice are presented in the Supplementary Figure S4b,c.

Unstimulated respiration (i) demonstrated subtle differences only when fueled by the palmitoyl carnitine (Figure 2, Supplementary Figure S4). Respiration stimulated by coupling to ATP synthesis (state 3) and respiration uncoupled from ATP synthesis by FCCP (state 3u) demonstrated a significant difference between the wild type and both *Mtln* knockout mice for both muscles and all substrates of the respiratory Complex I. Respiration on succinate, a substrate of the respiratory complex II, was found to be equally stimulated in the wild type and both *Mtln* knockout muscle mitochondria by ADP and FCCP in agreement with our earlier study on *Mtln* knockout cell lines [9].

### 2.5. Influence of Mtln on Creatine Shuttle System Functioning

Skeletal muscles use creatine/creatine phosphate (Cr/CrP) shuttling to facilitate diffusion of energy rich compounds from mitochondria to the sites of consumption (myosin filaments). For doing so, ATP, produced by mitochondria, is used to produce CrP from Cr. This reaction is catalyzed by mitochondrial creatine kinase (mtCK), an enzyme located in the mitochondrial intermembrane space and composed of either two (mtCK_2_) or eight (mtCK_8_) identical subunits. At the sites of energy expenditure, the reverse reaction takes place with the help of cytosolic CK, converting CrP to Cr coupled to ATP synthesis from ADP.

We tested the efficiency of respiration coupled to Cr phosphorylation (Figure 2, Supplementary Figure S4, lanes labeled “ADP (4) Cr”). Glycolytic tibialis anterior muscle mitochondria (Supplementary Figure S4a,c) do not reveal an efficient respiration coupling with Cr/CrP shuttle in both the wild type, Δ*Mtln-1* mice and Δ*Mtln-2* mice. For the oxidative soleus muscle mitochondria (Figure 2, Supplementary Figure S4b) we observed a difference between the wild type and both *Mtln* knockout mice respiration on the substrates of the Complex I (palmitoyl carnitine, pyruvate/malate, and glutamate/malate), which might be explained by a reduction in Complex I activity upon *Mtln* inactivation. However, we observed a significant reduction in succinate (Complex II substrate) dependent respiration for soleus muscle mitochondria of both *Mtln* knockouts if coupled to Cr phosphorylation, but not if respiration is uncoupled by FCCP (state 3u) or coupled with ADP phosphorylation (state 3). It appeared that the soleus oxidative muscle mitochondrial Cr/CrP shuttle depends on Mtln functionality.

**Figure 3:**
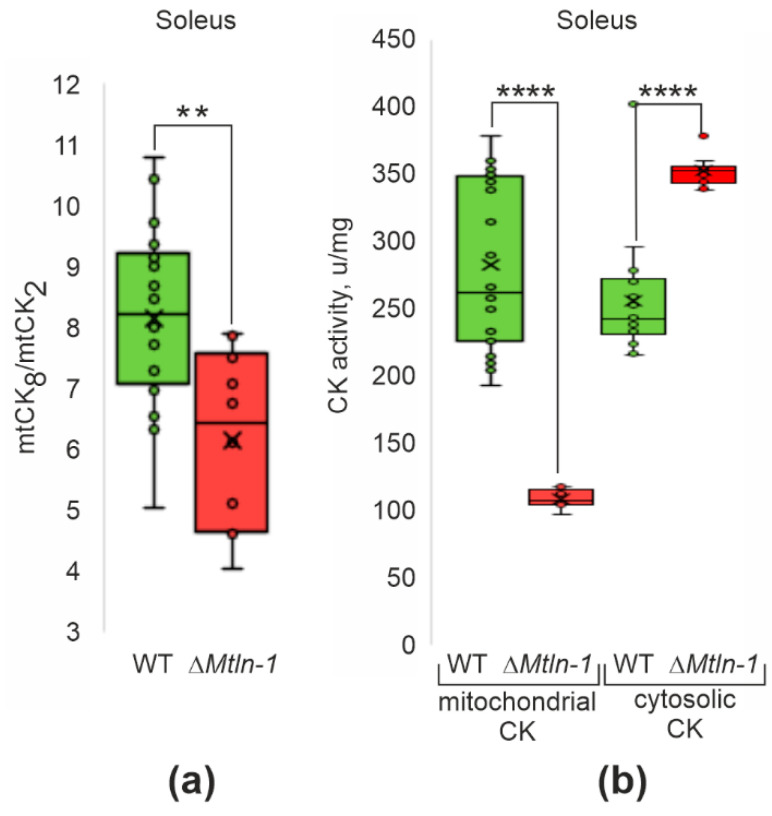
Influence of *Mtln* gene inactivation on creatine kinase functioning. (**a**) Relative abundance of the octameric and dimeric forms of the mitochondrial creatine kinase (mtCK) in the soleus muscle mitochondrial extracts of the wild type (green bars, *n* = 18) and Δ*Mtln-1* (red bars, *n* = 10) male mice; (**b**) Mitochondrial (left group of bars) and cytosolic (right group of bars) creatine kinase activity for the soleus extracts of the wild type (green bars, *n* = 18) and Δ*Mtln-1* (red bars, *n* = 10) male mice. For all panels, interquartile ranges are shown as solid bars, while the all-data range is shown by thin lines. The horizontal line corresponds to the median, while the cross is the average. Significance level calculated using the Student’s *t*-test is shown. If a *p*-value is less than 0.01, it is flagged with two stars (**), while four stars (****) corresponds to a *p*-value less than 10^−4^.

This result prompted us to address the oligomeric state and activity of the mtCK in the wild type and *Mtln* knockout mice lines. In both oxidative (soleus) muscle (Figure 3a, Supplementary Figure S5b,c) and glycolytic (tibialis anterior) muscle (Supplementary Figure S5a,b,d), we detected a significant reduction in the ratio of octameric to dimeric form of the mtCK upon *Mtln* inactivation.

Measurement of the mitochondrial CK activity in the oxidative muscle (soleus) for both Δ*Mtln-1* mice and Δ*Mtln-2* mice revealed a significant reduction upon inactivation of the *Mtln* gene (Figure 3b, Supplementary Figure S5g, left bars), while the activity of cytosolic CK enzyme (Figure 3b, Supplementary Figure S5g, right bars) and its amount (Supplementary Figure S5e) increased, which might represent a compensatory response. Quantitation of the mtCK in the soleus and tibialis anterior muscles of the wild type and Δ*Mtln-1* and Δ*Mtln-2* mice by immunoblotting (Supplementary Figure S5e) revealed a moderate, ca. 1.5-fold decrease in the mtCK amount in soleus muscles upon *Mtln* inactivation. Thus, a decrease in mtCK amount and reduction in mtCK octamerization, which is likely a consequence of cardiolipin exhaustion [21], both contribute to a decrease of mtCK activity. The same difference in the mitochondrial creatine kinase activity was not observed for the glycolytic (tibialis anterior) muscles (Supplementary Figure S5f,h), but in contrast we detected an increase in mtCK activity upon *Mtln* inactivation. This difference is likely to be explained by an increase in mtCK amount in the tibialis anterior muscles, which we detected (Supplementary Figure S5e) or by the difference in the major isoform of mtCK [22]. According to the published data [23], glycolytic muscles contain more ubiquitous umtCK, rather than sarcomeric smtCK, which differ in their properties, such as e.g., cardiolipin (CL) binding [24].

### 2.6. Influence of Mtln on Cardiolipin Structure and Content

**Figure 4:**
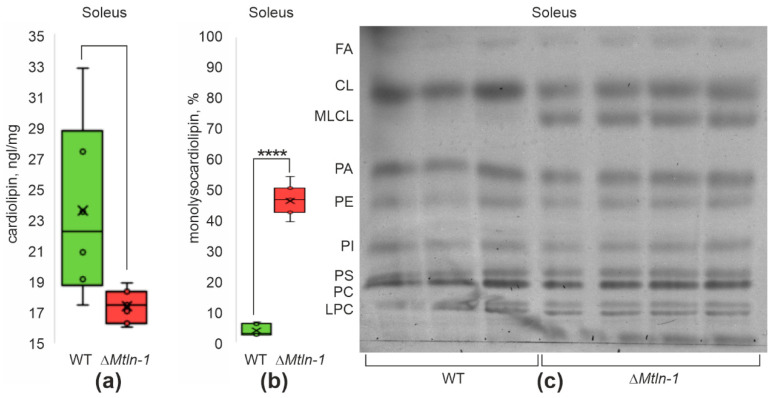
Influence of *Mtln* gene inactivation on cardiolipin amount and integrity. (**a**) Cardiolipin quantitation in the soleus mitochondria of the wild type (green bars, *n* = 6) and Δ*Mtln-1* (red bars, *n* = 7) mice; (**b**) Quantitation of the amount of monolysocardiolipin (MLCL) relative to the total amount of cardiolipin and monolysocardiolipin (MLCL+CL) in the soleus mitochondria of the wild type (green bars, *n* = 7) and Δ*Mtln-1* (red bars, *n* = 8) mice; (**c**) Thin layer chromatography of the soleus mitochondrial lipids of the wild type male (left 3 lanes) and Δ*Mtln-1* male (right 4 lanes) mice. Lipid designations are: fatty acids (FA), cardiolipin (CL), monolysocardiolipin (MLCL), phosphatidic acid (PA), phosphatidylethanolamine (PE), phosphatidylinositol (PI), phosphatidylserine (PS), phosphatidylcholine (PC), lysophosphatidylcholine (LPC). For panels a and b, interquartile ranges are shown as solid bars, while the all-data range is shown by thin lines. The horizontal line corresponds to the median, while the cross is the average. Significance level calculated using the Student’s *t*-test is shown. If a *p*-value is less than 10^−4^, it is flagged with four stars.

Earlier we demonstrated [9] that an influence of Mtln on the respiratory Complex I is indirect and likely to be mediated via modulation of lipid composition. Cardiolipin (CL) is the most specific lipid of the mitochondrial inner membrane needed to maintain cristae structure as well as an optimal functioning of a set of enzymatic complexes embedded as well as associated with the mitochondrial inner membrane, including Complex I [25,26]. Moreover, Stein and co-authors [10] demonstrated a direct interaction of Mtln with CL and the effect of this interaction on the stability of the respiratory chain complexes. Cardiolipin is known to interact with the mitochondrial creatine kinase (mtCK) and facilitate formation of the homooctameric complex of the latter from its homodimers [27], which is more prominent for the oxidative muscles [24]. We set out to test whether *Mtln* knockout influences cardiolipin concentration in the mitochondria purified from the oxidative (soleus) and glycolytic (tibialis anterior) muscle from the wild type and *Mtln* knockout mice (Figure 4a, Supplementary Figure S6a,d,f). In both types of muscles and both knockout lines, we revealed a significant drop in the cardiolipin concentration in mitochondria.

The main cause of cardiolipin exhaustion is prevalence of its oxidative damage above remodeling [28]. The *Mtln* gene inactivation was earlier found to increase reactive oxygen species (ROS) production [10]. As the major pathway of cardiolipin repair goes via damaged fatty acid excision [28], we monitored the monolysocardiolipin level in the soleus and tibialis anterior muscle mitochondria of both *Mtln* knockout mice lines (Figure 4b,c, Supplementary Figure S6b–f,h,j). In agreement with our expectation, we found a significant increase in the amount of monolysocardiolipin following *Mtln* gene inactivation in the mitochondria of both types of muscles.

## 3. Discussion

Several lines of evidence support an involvement of Mtln in a myogenic differentiation and physiological functioning of muscles [11,15,16] as the primary role of this peptide. However, the data on muscle performance upon *Mtln* inactivation are sometimes controversial. We observed a reduction in grip strength for *Mtln* gene knockout mice corroborating earlier reports [15,16]. Histological analysis revealed a tendency for increased regeneration of muscles in *Mtln* knockout mice, but lack of other signs of damage. We could not rule out, however, that some structural changes might have been revealed by other methods, such as, electron microscopy used by other groups [11].

Analysis of the soleus muscle transcriptome revealed only a minor influence of Mtln on the expression of individual genes. However, analysis of the overrepresentation of gene sets among up- and down-regulated genes in soleus muscles devoid of Mtln revealed significant down-regulation of ribosomal protein genes, both mitochondrial and cytosolic. Down-regulation of translation upon mitochondrial dysfunction has been observed before and is likely mediated by a decrease in mTOR signaling [29,30]. Among gene sets up-regulated upon Mtln gene inactivation, are those involved in monocarboxylic acids binding and arachidonic acid monooxygease activity.

**Figure 5:**
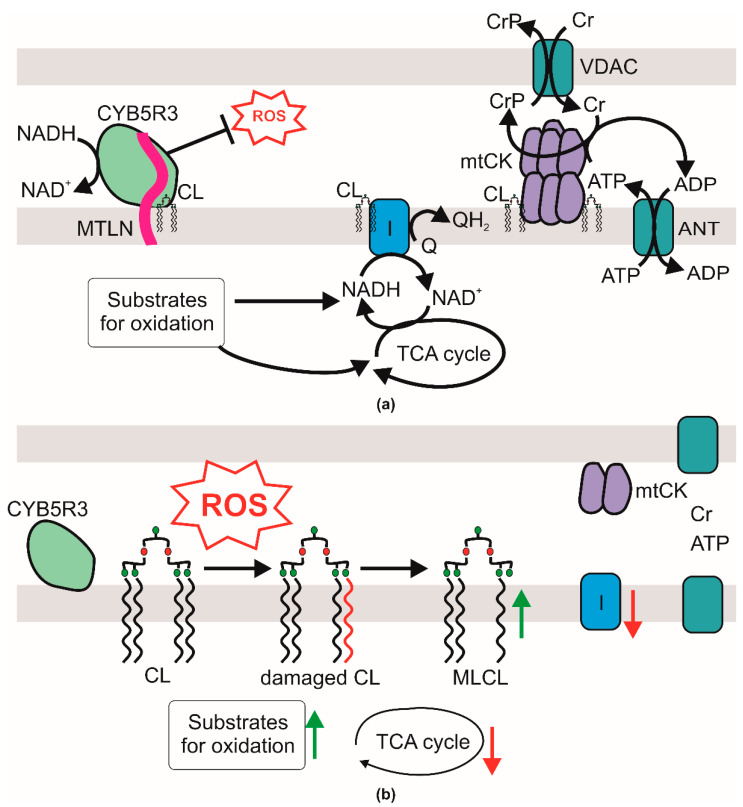
A model for the Mtln function. (**a**) Mitochondrial homeostasis in the presence of intact Mtln. Shown is an interaction of Mtln (pink) with cardiolipin (CL) and CYB5R3 NADH dehydrogenase preventing oxidative lipid damage by the reactive oxygen species (ROS). It ensures normal CL concentration and proper activity of the respiratory complex I (I). The tricarboxylic acids (TCA) cycle functions normally and ensures oxidation of substrates. Cardiolipin facilitates mitochondrial creatine kinase (mtCK) octamerization in the intermembrane space coupling ATP/ADP antiporter (ANT) activity, creatine (Cr) phosphorylation and creatine phosphate (CrP) export to the cytosol via VDAC. (**b**) Consequences of *Mtln* inactivation. Lack of Mtln leads to a loss of its interaction with CL and increased ROS production, likely due to the decreased CYB5R3 activity. Increased concentrations of ROS cause excessive CL damage. Monolysocardiolipin (MLCL), an intermediate of CL repair is accumulating. Decrease in the functional CL concentration leads to suboptimal function of CI which leads to the accumulation of substrates of respiration and depletion of its products and TCA cycle intermediates. Red downward arrows mark metabolites whose concentration is reduced or enzymes whose activity is reduced upon *Mtln* inactivation, while upward green arrows mark those metabolites whose concentration was increased. The CL concentration drop affects mtCK octamer formation and consequently the Cr/CrP dependent energy relay efficiency.

While molecular mechanisms linking Mtln peptide with the phenotypic manifestations of its knockout are still enigmatic, our study contributes to understanding of this process (see Figure 5 for the suggested model). It was earlier demonstrated that Mtln interacts with Cyb5r3 NADH dehydrogenase involved in fatty acids metabolism [31,32,33] as well as in the prevention of lipid oxidative damage and enhancement of respiratory complex functioning [34]. Likewise, inactivation of *Mtln* was shown to increase ROS production [10]. Cardiolipin, a unique mitochondrial lipid which is practically indispensable for respiratory chain CI function [25], is one of the major targets for ROS-induced lipid damage [28]. On the basis of the results presented in this work, we hypothesize (Figure 5) that the observed decrease in the concentration of cardiolipin might be explained by its excessive oxidative damage caused by a decrease of Mtln-dependent protective function of Cyb5r3. Apart from Cyb5r3 mediated CL protection, we could not exclude a direct protection of CL by Mtln, which might be mediated by an interaction of these molecules shown by Stein and co-authors [10]. Previously, an interaction of Mtln with mitochondrial trifunctional protein was described [11]. Direct involvement of the HADHA subunit of the trifunctional protein in CL remodeling [35] might provide another possible link between Mtln and CL homeostasis. Preferential removal of a damaged fatty acid from the CL molecule by Ca^2+^-independent inducible mitochondrial phospholipase A2γ (iPLA2γ) leads to the accumulation of MLCL, which we observed here, and can play a pivotal role in production of oxylipins—essential second-messengers linking mitochondrial bioenergetics and intracellular signaling during oxidative stress [36,37]. Whereas oxylipins are substrates of DHRS9 [38], whose expression was found increased in our study, it is interesting to point out that Cyb5r3 is also involved in Δ9 desaturase activity, so a potential influence of *Mtln* inactivation on Cyb5r3 activity can be compensated by a DHRS9 increase.

The difference in serum free fatty acids concentrations, i.e., a decrease in the amount of saturated fatty acids and increase in the amount of polyunsaturated DHA observed for Δ*Mtln* mice [17] is reminiscent of the accumulation of DHA-containing triglycerides in the cell devoid of Mtln [9]. Saturated fatty acids are known to be reluctant to be involved in CL repair and are incorporated into CL preferentially during its biogenesis [39], while DHA content increases in CL with age [40] and obesity [41] negatively influencing respiration. The molecular mechanisms connecting Mtln function in cardiolipin protection and fatty acids β-oxidation and observed fatty acids disbalance might be a topic for a further study.

Repair of oxidized cardiolipin by Tafazzin (TFZ) consumes phosphatidyl choline whose concentration decrease we observed earlier [9]. Apart from the CI, cardiolipin influences mitochondrial creatine kinase [27]. In line with this, we observed decay of mitochondrial creatine kinase octamer into dimers which leads, in the case of oxidative muscles, to the decrease in the mitochondrial creatine kinase activity and creatine shuttle efficiency, which we assume is a consequence of CL concentration decrease (Figure 5). Previously, such a combination of the observed phenomena has already been noted in mice deficient in creatine kinase in a study where the effect of the absence of the mitochondrial or cytosolic creatine kinase gene or both genes was investigated (reviewed in [42]). Indeed, contact sites between inner and outer mitochondrial membranes mediated by an octamer of mtCK plays an essential role in cardiolipin transfer between mitochondrial membranes and MAM/ER during lipid maturation and remodeling by the external enzyme ALCAT-1 (Acyl-CoenzymeA:Lysocardiolipin Acyltransferase-1) with specificity to poly-unsaturated acyls [43]. Disruption of creatine kinase octamers abolishes the trend to increase PUFA-CL and reduces the probability of formation of lipid peroxidation products upon enhanced ROS but also reduction in PUFA-CL leads to decrease in respiration efficacy [43].

Suboptimal efficiency of respiratory chain CI results in the accumulation of substrates for oxidation or their precursors [17], such as triglycerides and amino acids and depletion of the intermediates of the TCA cycle, such as citrate, succinate and malate as well as other products of oxidation, such as betaine (Figure 5).

With each study devoted to the Mtln peptide, its role has become increasingly clear. While the model (Figure 5) presented on the basis of this study is still an approximation with many assumptions, it creates a framework for an understanding of Mtln molecular function.

## 4. Materials and Methods

All manipulations were conducted in compliance with the protocol approved by the Local Bioethics Commission of the Research Center “Institute of Mitoengineering of Moscow State University” LLC, (Moscow, Russia), Commission decision № 79 dated July 2015, № 133 dated 23 April 2018, the Bioethics Commission of Lomonosov MSU № 76 dated 10 May 2018. Whenever possible, the same animals were used for several experiments in accordance to the bioethics demand on “Reduction” (decreasing the overall numbers of animals used).

Inactivation of the *Mtln* gene was done using the CRISPR/Cas9 system using the standard procedure [44] as described previously [17]. All experiments were performed on year-old male mice to avoid an excessive data dispersal due to the differences in the stage of estrous cycle. We expect that the general outcome of the experiments would be similar for female animals as well but might require more animals to reach statistical significance.

The grip force test was done according to [45]. The electrostimulated contraction of the tibialis anterior, soleus and gastrocnemius muscles were assessed by an Electronic Laboratory Stimulator (ESL-2, Moscow, Russia). Mitochondria of soleus and tibialis anterior muscles were prepared as described [46]. The rate of oxygen consumption was measured using a closed-type Clark electrode on Hansatech oxygraph (Norfolk, UK) as described before [46]. The creatine kinase activity was measured spectrophotometrically in a coupled assay with hexokinase and glucose-6-phosphate dehydrogenase and ATP/phosphocreatine as substrates. Dimeric and octameric mitochondrial creatine kinase isoforms were separated by cellulose-polyacetate-gel electrophoresis and detected enzymatically.

More details on the study methodology are provided in Supplementary Methods.

## Data Availability

Results of transcriptome sequencing are available in SRA via accession PRJNA844239.

## Supplementary Materials

The following supporting information can be downloaded at https://www.mdpi.com/article/10.3390/ijms24087589/s1.
