# Supplementary material for: Mitoregulin Contributes to Creatine Shuttling and Cardiolipin Protection in Mice Muscle
Source: Int J Mol Sci. 2023 Apr 20;24(8):7589. doi: 10.3390/ijms24087589 (PMC10143810; doi:10.3390/ijms24087589)
Supplement: Supplementary file 1 [file ijms-24-07589-s001.zip › SI Averina on template v3.pdf]

Supplementary materials

Mtln contributes to creatine shuttling and cardiolipin protection in mice muscle

Olga A. Averina[1,2], Oleg A. Permyakov[1], Mariia A. Emelianova[3], Olga O. Grigoryeva[1], Maxim L. Lovat[2,4], Anna E. Egorova[5], Andrei V. Grinchenko[6], Vadim V. Kumeiko[5,6], Maria V. Marey[7], Vasily N. Manskikh[2,4], Olga A. Dontsova[2,3,8,9], Mikhail Yu. Vyssokikh[2,7*] and Petr V. Sergiev[1,2,3,8*]

[1] Institute of Functional Genomics, Lomonosov Moscow State University, 119992, Moscow, Russia
[2] Belozersky Institute of Physico-Chemical Biology, Lomonosov Moscow State University, Moscow, 119992, Russia
[3] Center for Life Sciences, Skolkovo Institute of Science and Technology, Skolkovo, Moscow region 143025, Russia
[4] Institute of Mitoengineering MSU, 119992, Moscow, Russia
[5] Far Eastern Federal University, 690922, Vladivostok, Russia
[6] A.V.Zhirmunsky National Scientific Center of Marine Biology, 690041, Vladivostok, Russia
[7] Research Center for Obstetrics, Gynecology and Perinatology, 117198, Moscow, Russia
[8] Department of Chemistry, Lomonosov Moscow State University, Moscow, 119991, Russia
[9] Shemyakin-Ovchinnikov Institute of Bioorganic Chemistry, Russian Academy of Sciences, Moscow 119992, Russia
* Correspondence: mikhail.vyssokikh@gmail.com (M.Y.V.); petya@genebee.msu.ru (P.V.S.)

1. Supplementary materials and methods

1.1 Animal handling

All manipulations were conducted in compliance with the protocol approved by the Local Bioethics Commission of the Research Center "Institute of Mitoengineering of Moscow State University" LLC, (Moscow, Russia) (http://www.vec-msu.ru/), Commission decision №79 dated July 2015, № 133 dated April 23, 2018, the Bioethics Commission of Lomonosov MSU № 76 dated May 10, 2018. All experiments were performed on year-old male mice.

The animals were kept in individually ventilated cages (IVC system, TECNIPLAST S.p.A., Italy), with unrestricted access to food and water, in an environment free of specific pathogens, under a 12:12 h light/dark cycle, 35 lux. Wild type C57BL/6J and *Mtln* knockout mice on the same genetic background were obtained as described [1]. Mice were genotyped by genomic DNA amplification with the primers GAGTCAGGGAACTCTGCTTCCTTT and CTCAGGCCAGGTCCAGCTTTTTC followed by Sanger sequencing (Center of Collective Use «Genome» at Engelhard Institute of Molecular Biology, Moscow, Russia).

1.2 Mtln gene genotypes

In this work we used two *Mtln* knockout mice lines. All experiments have been done with the *ΔMtln*-1 line containing 82 nt. deletion (CTCAAGCGAGCGCTGTGGCATCTGATCCTTGCGCCAATCCGTAGCTCACTCTACTTTGTGCTGAGTGGTTGCA**ATG**GCGGAC) encompassing *Mtln* start codon (shown in bold) and promoter region which we described previously [1]. In addition, to confirm major results, namely, mitochondrial respiration on a complete set of substrates, creatine kinase oligomeric state and activity and cardiolipin and monolysocardiolipin quantitation we used an independently generated *ΔMtln*-2 line carrying 8 nt. deletion (GTTGCA**ATG**) eliminating *Mtln* start codon (shown in bold). This *Mtln* Δ8 line has been produced identically to the Δ82 line described, but was originated in a different founder.

Both lines have been backcrossed 3 times to C57Bl/6J line. Heterozygous carriers of inactivating alleles have been mated and obtained homozygous progeny were used to establish Δ*Mtln-1 (*Δ82/Δ82*)* and Δ*Mtln-2 (*Δ8/Δ8*)* knockout and wild type control lines for all further experiments. Potential influence of genetic background beyond *Mtln* gene has

been minimized as the control wild type mice line used originated from the littermates of knockout mice in a cross of heterozygous knockout allele carriers.

1.3 Grip strength

The test is designed to evaluate the maximum voluntary strength (grip force), and is a common method of mice and rats limb strength and neurological deficit determination [2]. The experiment was performed after 24 hours food deprivation. Mouse pulls gently the back by its tail ensuring the mouse grips the top portion of the grid and the torso remains horizontal and record the maximal grip strength value of the mouse that is displayed on the screen. This procedure is repeated with 15-30 s range until 10 results obtention. After selection of five maximum obtained values the weight normalized mean value is calculated.

1.4 Electrostimulated contraction of muscle

Tibialis Anterior (TA), Soleus (Sol) and Gastrocnemius (Gas) muscles were sequentially isolated according to the standard technique [3,4]. The muscle distal end was connected through a ligature to a mechano-electronic transducer (MET), the proximal end was secured with a clamp. Muscle contractions stimulating and recording unit included an Electronic Laboratory Stimulator (ESL-2, Russia) and a pair of electrodes with fasteners attached to the muscle (direct stimulation). The electrostimulator allowed to change the amplitude of the stimulus, its duration and the frequency of supplied excitations. The signal of strain gauge measuring muscle contraction strength was amplified on a computer, that allowed to register muscle contractions and their strength in grams using an analog-to-digital converter (ADC E-154, Russia) and the PowerGraph 3.3 software (DiSoft, Russia). The strain gauge was matched specifically based on the need to register the mouse muscle contractions strength with an accuracy of 0.01 g. The muscle was stimulated with rectangular pulses (amplitude 20 V, duration 0.5 ms). The impulse amplitude selected was twice the level of maximum contraction observed, which ensured the involvement of all muscle motofacient units. The pulse frequency in the burst was 40 Hz, which corresponds to smooth tetanus mode stimulation. This mode was selected to assess the anaerobic resource of the muscle: the maximum amounts of calcium ions are released from the muscle fibers SPR with a stimulus of 40 Hz leading to a constant ATP and creatine phosphate stocks reduction and consumption, accumulation of metabolic products and depletion of muscle energy reserves normally accumulated through glycolysis. The muscle was weighed after mechanogram recording for mouse strength-per-weight rationing.

1.5 Histology

Male and female kidneys, abdominal fat samples, spleens, livers, tibialis anterior and soleus muscles were used for histopathological examination. The specimens were fixed with 10% buffered formalin solution (pH 7.4), trimmed, dehydrated with 99.7% isopropanol, and paraffin-embedded. Microtome sections (3 μm) were deparaffinized, hydrated, and stained with hematoxylin and eosin. Pathologies were diagnosed and classified according to published recommendations [5,6].

For study of myopathy lesions, fresh samples of skeletal muscles were sectioned with cryotome. Unfixed cryotome sections with thickness of 5 μm were stained with Gomori trichrome [7] for elucidation of myofibers with signs of mitochondrial abnormalities. Sections were stained with filtered hematoxylin for 5 minutes, washed with distilled water, stained with filtered Gomori trichrome stain (Fast Green FCF 0.3 g, Chromotrope 2R 1.2 g, Phosphotungstic acid 0.6 g, Glacial acetic acid 1 ml, distilled water 100 ml, pH 3.4) for 15 minutes, washed with distilled water and rinse briefly in 0.5% acetic acid for

differentiation. Stained sections were dehydrated with 95% ethanol, cleared with xylene and mounted.

1.6 Tissues processing, mitochondria preparation and respiration analysis

Soleus or tibialis anterior muscles of wild type and mutant mice were excised, trimmed free of sheath and fascia, weighed and placed in ice cold isolation medium (see below). Same animals were used to process soleus and tibialis anterior. Part of the muscle (5–10 mg) was immediately frozen in liquid nitrogen and stored at -80°C for later determinations of enzyme activities, oligomer state of creatine kinase and creatine concentration. Mitochondria preparation from skeletal muscle of wild type and mutant mice was done as described before with slight modifications [8]. For that tissue fragments were gently minced by small cooled scissors into 0.5 mm pieces and due to the small size of samples homogenized in homemade 0.5 ml Potter Teflon–glass microhomogenizer with 200 micron clearance in 10 volumes (v/w) of 250 mM sucrose, 0.5 mM EGTA, 20 mM HEPES-NaOH, pH 7.6, and 0.1% BSA (isolation medium) for 2 min at 4°C, with a ratio of 5/1 volume to weight of tissue fragment. The homogenate was centrifuged at 1000 g for 10 min at 4°C in a centrifuge (5410 Eppendorf, Germany). The supernatant was collected and centrifuged at 9000 g under the same conditions. Mitochondrial pellets were collected and suspended in the same volume of isolation medium lacking BSA (a microhomogenizer and centrifugation at 10,500 g for 10 min at 4°C were applied). The resulting pellet was suspended in a minimal volume (approximately 1ul/mg of initial tissue weight) with a typical concentration of 90-100 mg/mL, as determined using the bicinchoninic acid method with BSA as the standard, according to the manufacturer's instructions (Pierce, USA). All procedures were performed in a cold box at 4°C.

To assess the respiration capacity of the isolated mitochondria, the rate of oxygen consumption was measured at 25 ° C using a closed-type Clark electrode on Hansatech oxygraph (Great Britain) as described before [8]. Mitochondria (0.05-0.1 mg protein) were incubated in an oxygraph cell containing 0.5 mL of MIR05 [9] respiration medium (EGTA 0.5 mM, 3 mM $MgCl_2$, 60 mM potassium lactobionate, 20 mM taurine, 10 mM KH_2PO_4, 20 mM HEPES, 110 mM sucrose, 1 g/L BSA) and the efficiency of respiration was evaluated in the presence of 10 mM palmitoyl L-carnitine, 5 mM glutamate/1.25 mM malate, 5 mM pyruvate/1 mM malate or 5 mM succinate/ 2 μM rotenone. Then indicated 1 μM oligomycin or 0.1 mM ADP or 0.1 mM ADP/25 mM creatine or 10 nM FCCP was added.

1.7 Creatine kinase activity and oligomerization assay

An aliquot of muscle tissue homogenate (nuclei free supernatant after 10 min at 1000g) prepared as described above for mitochondrial isolation was used to measure creatine kinase activity and to determine the octamer/dimer ratio. Creatine kinase (EC 2.7.3.2) activity was measured in agreement with Bucher et al. [10]. Enzyme activity was determined in a coupled optical enzyme assay at 340 nm performed at Cary Varian 300 dual spectrophotometer with hexokinase from yeast and glucose-6-phosphate dehydrogenase from *Leuconostoc mesenteroides* (Roche) as coupling enzymes at pH 7.5 and ATP/phosphocreatine as substrates.

For detection of dimeric and octameric mitochondrial creatine kinase isoforms cellulose-polyacetate-gel electrophoresis was used. Octamer and dimer due to its different charge were separated electrophoretically (tissue extracts volumes was 1-5 μl) on cellulose polyacetate strips and Gelman Sepraphore III (USA) under non-denaturing conditions in 0.06 M Veronal buffer (pH 8.6) containing 1 mM 2-mercaptoethanol for 3 h at 250 V and stained for enzyme activity *in situ* as described [11]. Staining for CK activity was achieved by the coupled enzymes system (see above) and overlay-gel technique. Staining due to myokinase activity was inhibited by presence of 0.3 mM diadenosine pentaphosphate. Quantification of stain intensity was performed by densitometry in bands relevant to

octamer or dimer on ChemiDoc (Biorad, USA). Calibration of bands intensity was done with use of dimeric creatine kinase from rabbit muscle (Roche).

1.8 Lipid analysis

Lipids extraction was performed according to the method of Bligh and Dyer under a stream of nitrogen with oxygen free solutions bubbled with N_2 [12]. Extracted lipids were solved in chloroform/methanol mixture 2:1 (v/v). Thin layer chromatography was done according to the published procedure [13]. Analytical grade organic solvents, HPTLC chromatography plates (20 × 10 cm silica gel 60 F254 aluminium plates) were obtained from E. Merck (Darmstadt, Germany). Before sample or standard application HPTLC plates were prepared by immersion to 2.3% boric acid in ethanol, and then dried for two hours under fume hood and activated at 110 °C for 20 min on sand bath.

Samples were applied with the homemade glass capillary sample applicator with valve (driven with nitrogen stream) as 10 mm-long bands, 15 mm from the bottom of the plate, at a constant application rate about 200nL/s, under continuous drying with a stream of nitrogen at 4 bars. For PLs standards (Avanti Polar Lipids, France), a stock solution (1 mg/mL) was prepared in chloroform/methanol (2:1, v/v). Elution was done in a glass chamber equilibrated with vapor of eluent for 1 hour; eluent consisted of a mixture of chloroform/ethanol/triethylamine/water (3/3.5/3.5/0.7, v/v).

After a 1D development and two hours drying under fume hood, staining was performed by immersion of plates in to 0.5% copper sulphate (w/v) in 1.16 M orthophosphoric acid for 2 min, plates were dried at fume hood for 2 hours at room temperature and heated at sand bath for 15 min at 155 °C to carbonize organic matter and visualize PLs.

Plates were photographed in reflection mode under white light in the Biorad ChemiDoc. Images were analyzed in absorbance mode. PL spots intensities were integrated and peaks surfaces were expressed in arbitrary units with help of Image J and after calibration with CL and MLCL standards converted to nmoles/mg of mitochondrial protein.

1.9 Whole transcriptome assay

Transcriptome libraries were prepared using mRNA isolated by polyA fractionation and Dynabeads® mRNA DIRECT™ Micro Kit (Invitrogen, USA) from soleus muscle of the wild type (n=3) and *Mtln* knock-out (n=3) mice. The quality and concentrations of libraries were measured by automated electrophoresis system Agilent 4200 Bioanalyzer™ with the High Sensitivity ScreenTape kits (Agilent, USA) and Qubit 4 fluorometer (Thermo Fisher Scientific, USA), respectively. The libraries were proceeded in three replicas by Ion Total-RNA Seq Kit v2 according the manual provided. Chip loading was done using Ion Chef™ Instrument with the Ion 540™ Chef Kit (Thermo Fisher Scientific, USA), while sequencing was performed on Ion GeneStudio™ S5 System (Thermo Fisher Scientific, USA).

Read quality was analysed by the FastQC program. Reads with unsatisfactory quality and/or length were removed utilizing the Trimmomatic-0.36 package. The reads were aligned from the obtained files to the reference genome of Mus musculus with the assembly GRCm39 (GCA_000001635.9) by the STAR 2.7 software [14]. The resulting files with reads aligned and sorted by coordinates were used to obtain the count matrix by means of the HTSeq package. The obtained count matrix was analyzed using the web application Phantasus, integrated into the R environment. Differentially expressed genes were evaluated using the Limma package. Adjustment p-values (q-value or FDR, false discovery rate) for genes were set at less than 0.05 to detect differentially expressed ones. Functional enrichment analysis was performed with GSEA [15] against gene onthology (GO) molecular function database [16].

1.10 Antibody used

Immunoblotting have been done with anti-Mtln custom made rabbit antibodies against CRRLQDKLATTQKKLDLE peptide produced by Eurogentec S.A., mouse anti-GAPDH antibody (39-8600, clone ZG003, ThermoFisher Scientific), mouse anti-mtCK antibody (ab131188, Abcam), mouse anti-cytosolic CK antibody (ab72004, Abcam), rabbit anti-VDAC1 antibody (ab34726, Abcam) and rabbit anti-tubulin α antibody (ab18251, Abcam).

2. Supplementary figures

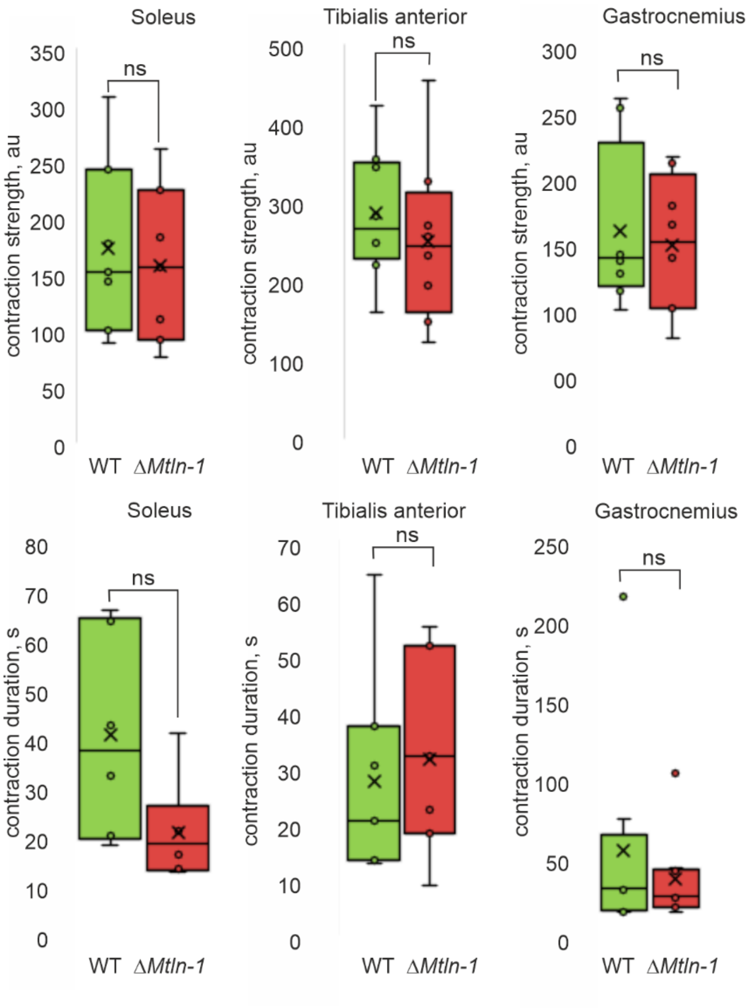

Supplementary Figure S1. Muscle contraction strength and duration upon electrostimulation after 24 hours food deprivation. Contraction strength related to the weight of mice and duration (seconds) of the oxidative soleus, glycolytic tibialis anterior and mixed gastrocnemius muscles of the wild type male (green bar, n=8) and Δ*Mtln-1* male (green bar, n=8) mice upon electrostimulation. Muscle identities are shown above the panels. For all panels interquartile ranges are shown as solid bars, while all data range by thin lines. Horizontal line corresponds to median, while cross to the average. Significance level calculated accordingly to the Student's t-test is shown.

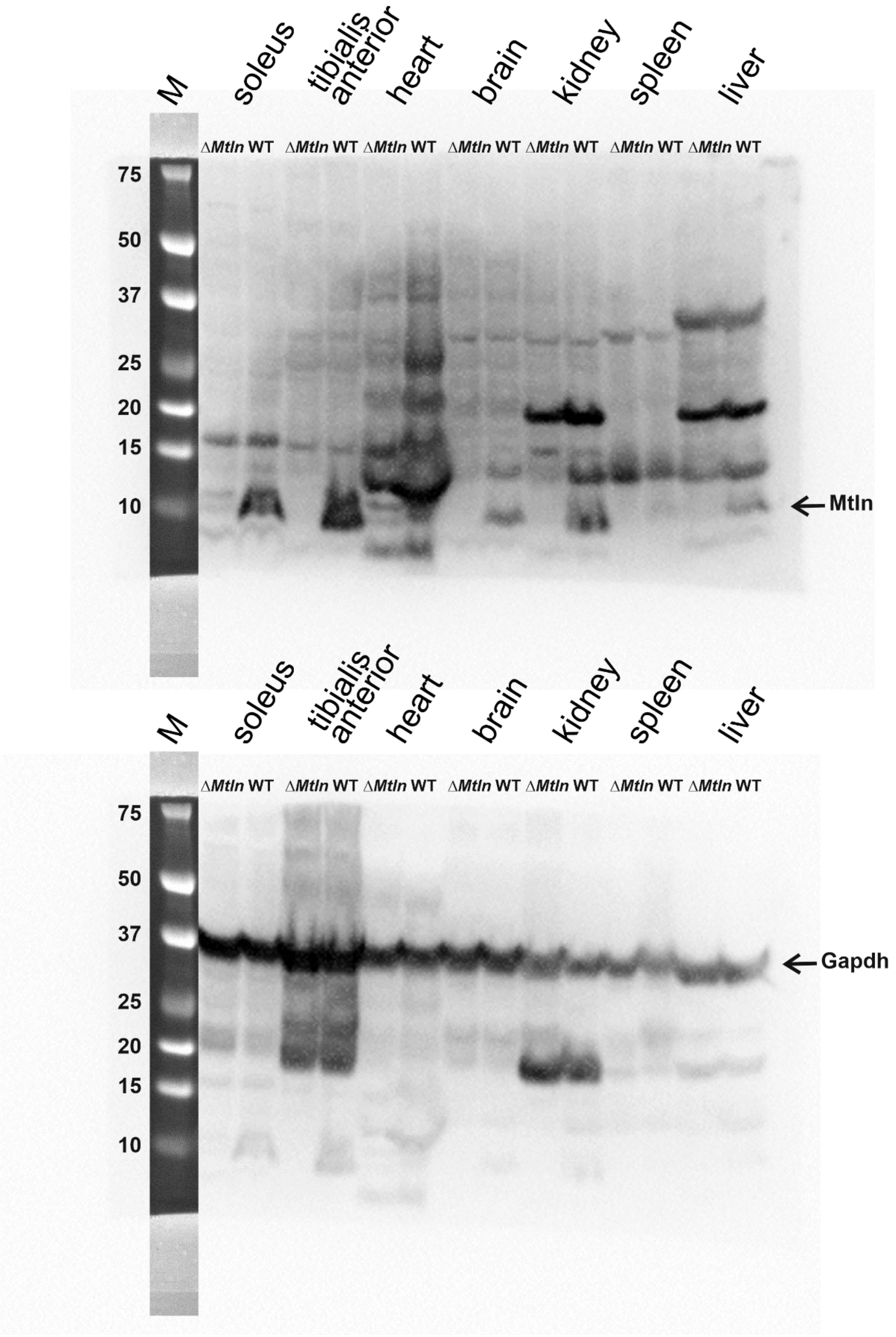

Supplementary Figure S2. Tissue distribution of Mtln. Immunoblotting of the WT (right lanes in each pair) and *ΔMtln-1* (left lanes in each pair) mice organs labeled above the lanes with anti-Mtln (upper panel) and anti-GAPDH (lower panel, same gel after anti-Mtln antibody stripping was used) antibodies. Marker proteins lane is designated (M). The bands corresponding to Mtln and GAPDH are marked by arrows.

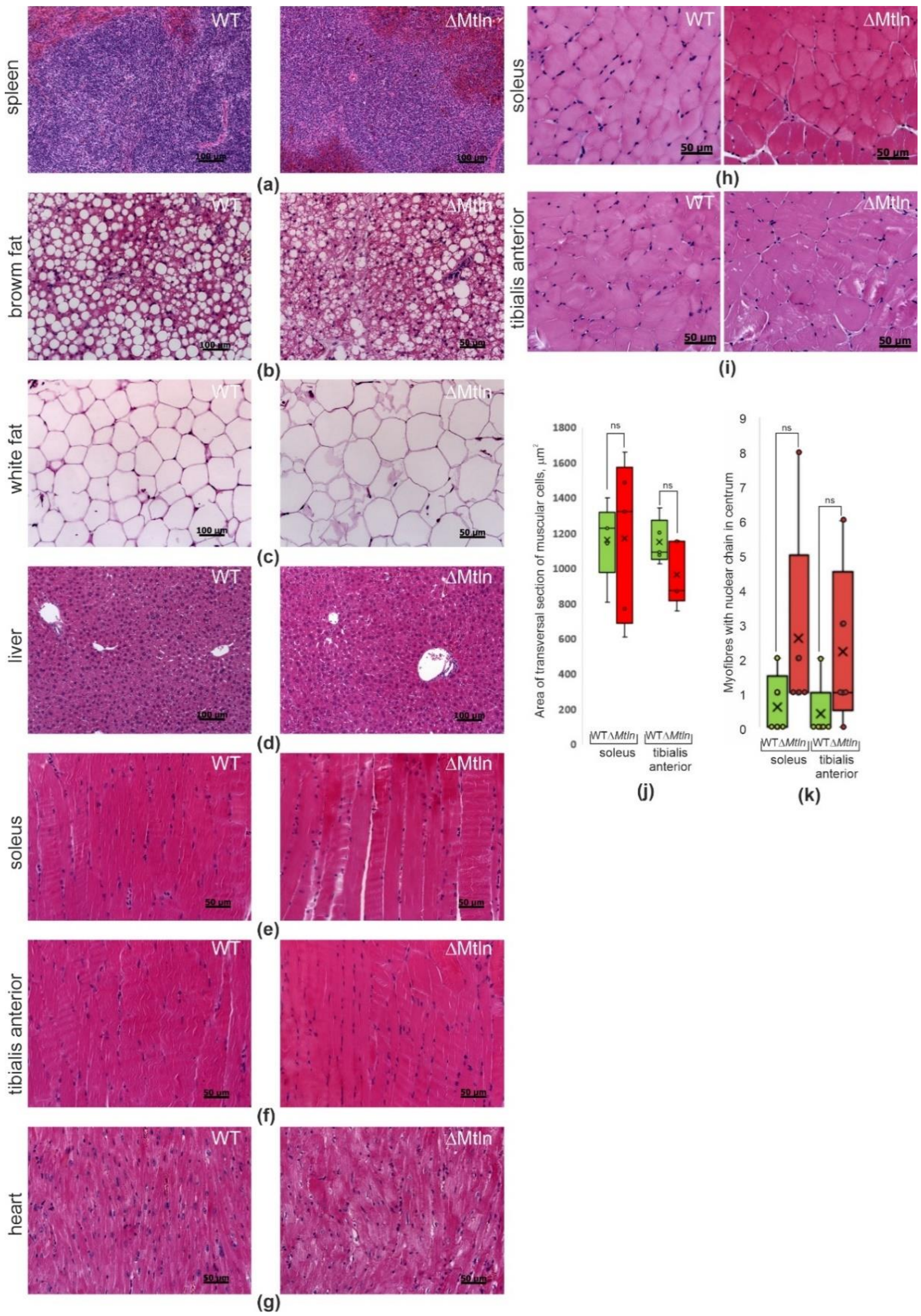

spleen
brown fat
white fat
liver
soleus
tibialis anterior
heart
WT
ΔMtln
100 µm
50 µm
(a)
(b)
(c)
(d)
(e)
(f)
(g)
(h)
(i)
(j)
(k)
Area of transversal section of muscular cells, µm²
Myofibres with nuclear chain in centrum
ns
WT ΔMtln
soleus
tibialis anterior

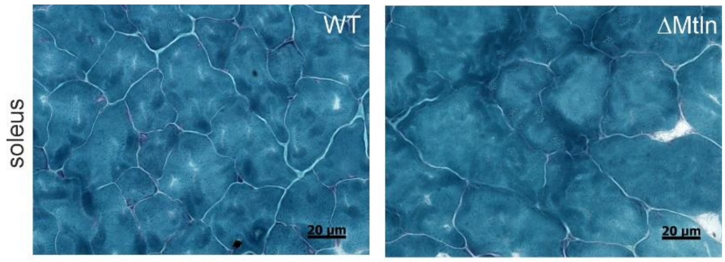

(l)

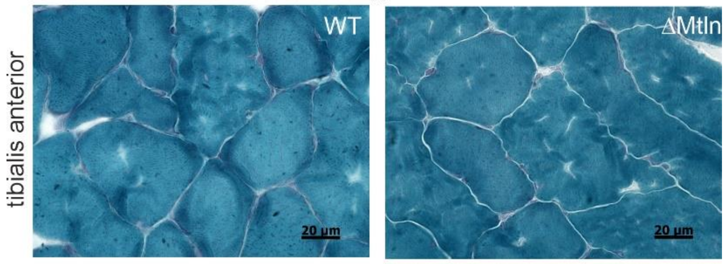

(m)

Supplementary Figure S3. Histopathological analysis of the WT and Δ*Mtln-1* knockout male mice. (**a-g**) Histopathology of the wild type (left panels) and Δ*Mtln-1* knockout (right panels) mice. Hematoxylin/eosin staining used throughout the panels. Tissue types marked above the panels; (**h,i**) Cross-fiber sections of soleus (**h**) and tibialis anterior (**i**) muscles of the wild type (left panels) and Δ*Mtln-1* knockout (right panels) mice. (**j**) Quantitation of the transversal area of soleus (left group of bars) and tibialis anterior (right group of bars) muscle cells (n=50 cells per mouse) of the wild type (green bars, n=5), Δ*Mtln-1* (red bars, n=5) mice; (**k**) Quantitation of the myofibers with nuclear chain in the center of soleus (left group of bars) and tibialis anterior (right group of bars) muscle cells (n=150 cells per mouse) of the wild type (green bars, n=5), Δ*Mtln* (red bars, n=5) mice. Interquartile ranges are shown as solid bars, while all data range by thin lines. Horizontal line corresponds to median, while cross to the average. Significance level calculated accordingly to the Student's t-test; (**l,m**) Assessment of damaged mitochondria in the soleus (**l**) and tibialis anterior (**m**) muscle of the wild type (marked) and Δ*Mtln-1* (marked) mice. Damaged mitochondria would be stained red.

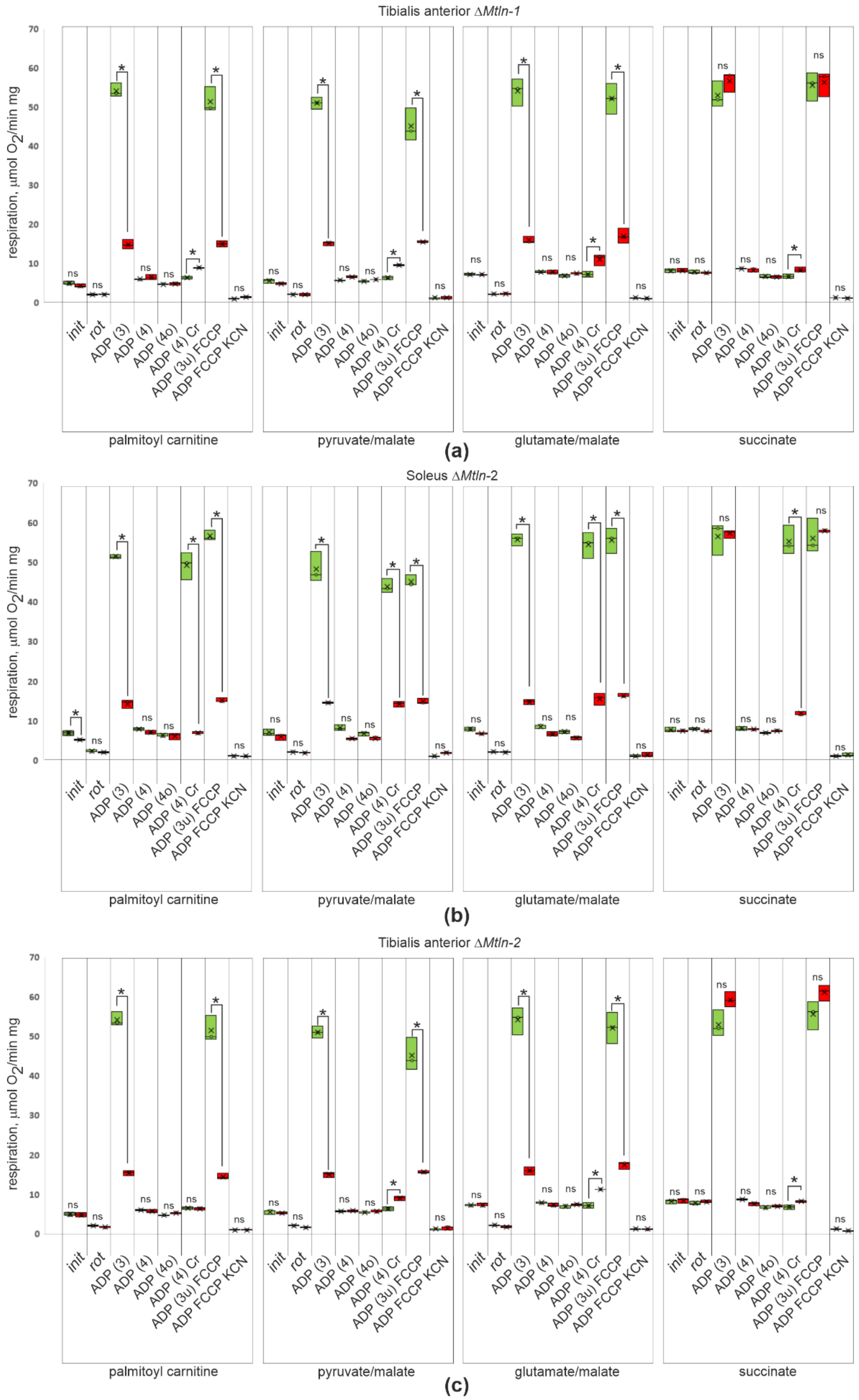

Supplementary Figure S4. Influence of *Mtln* gene inactivation on respiration of muscle mitochondria (related to the main text Figure 2). (**a**) Oxygen consumption rate (OCR) of tibialis anterior muscle mitochondria extracted from the wild type (green bars, n=3) and Δ*Mtln-1* (red bars, n=3) male mice. The groups of bars correspond to the respiration on palmitoyl carnitine (CI+CII+ETF activity), pyruvate and malate (CI activity), glutamate and malate (CI activity) and succinate (CII activity) as marked below the graphs. The experimental points measured are substrates alone (init), substrates

with rotenone (rot), substrates and ADP (ADP (3)), substrates after exhaustion of ADP (ADP(4)), substrates after exhaustion of ADP after addition of oligomycin (ADP (4o)), substrates after exhaustion of ADP (ADP(4)) following addition of creatine, substrates after exhaustion of ADP after addition of oligomycin and FCCP uncouper (ADP (3u) FCCP), residual respiration after inhibition of uncoupled respiration by cyanide. (**b**,**c**) Oxygen consumption rate (OCR) of soleus and tibialis anterior muscle mitochondria extracted from the wild type (green bars, n=3) and Δ*Mtln-2* (red bars, n=3) male mice. Designations similar to (**a**). Interquartile ranges are shown as solid bars, while all data range by thin lines. Horizontal line corresponds to median, while cross to the average. Significance level calculated accordingly to the Student's t-test is shown.

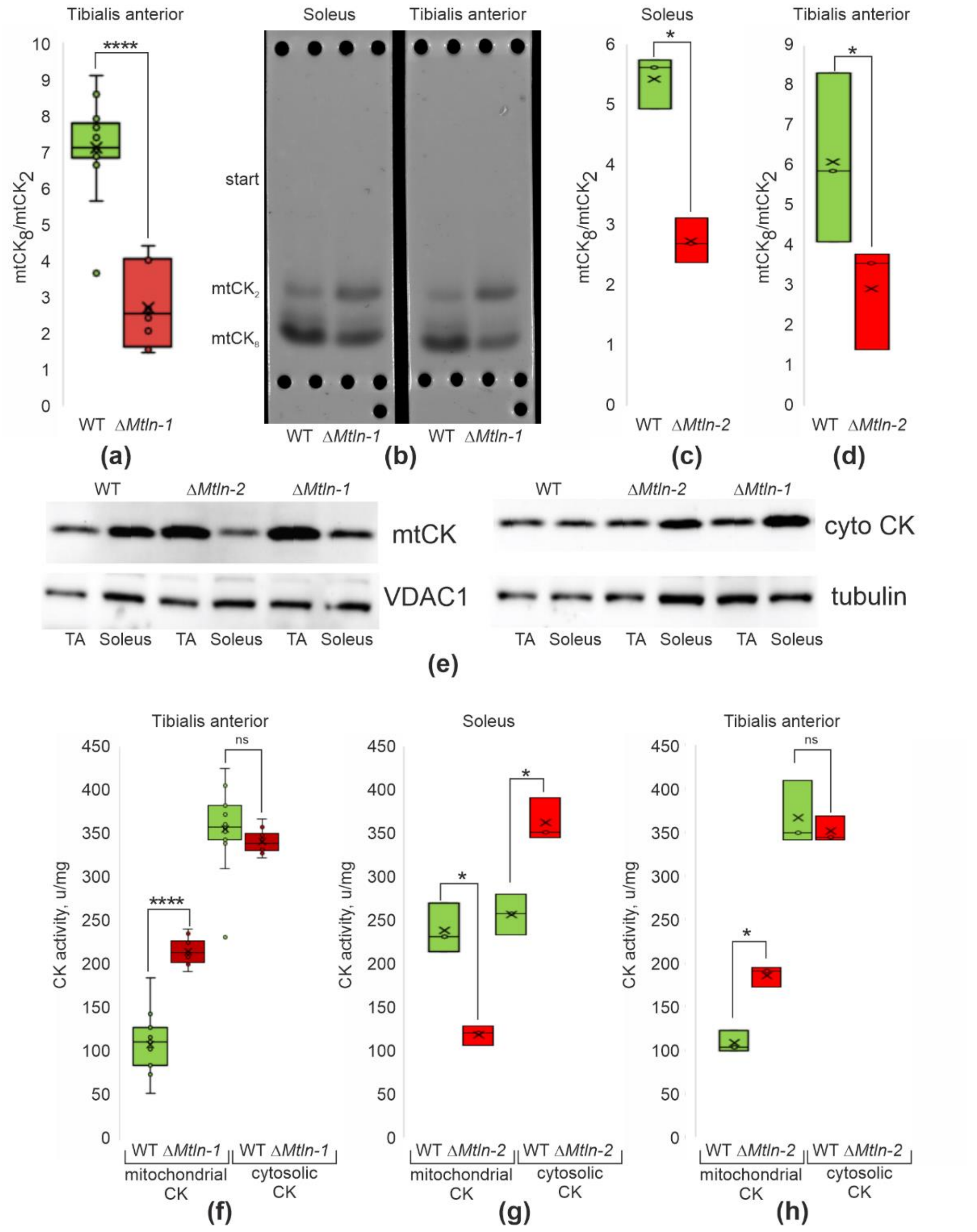

Supplementary Figure S5. Mtln influence on creatine kinase shuttle (related to the main text Figure 3). (**a**) Relative abundance of the octameric and dimeric forms of the mitochondrial creatine kinase (mtCK) in the tibialis anterior muscle mitochondrial extracts of the wild type (green bars, n=18) and Δ*Mtln-1* (red bars, n=10) male mice; (**b**) Detection of oligomer forms of mtCK by native cellulose polyacetate electrophoresis. Extracts from soleus and tibialis anterior mitochondria of the wild type and Δ*Mtln-1* knockout mice are used for separation. Creatine kinase is stained enzymatically; (**c**,**d**)

Relative abundance of the octameric and dimeric forms of the mitochondrial creatine kinase (mtCK) in the soleus and tibialis anterior muscle mitochondrial extracts of the wild type (green bars, n=3) and Δ*Mtln-2* (red bars, n=3) male mice; (**e**) representative mtCK (upper left panel) and cytosolic CK (upper right panel) quantitation by immunoblotting in the soleus and tibialis anterior (TA) muscle extracts of the wild type (WT, n=3), Δ*Mtln-1* (n=3) and Δ*Mtln-2* (n=3) male mice. Lower panels demonstrate loading controls (VDAC1 and tubulin) staining; (**f**) Mitochondrial (left group of bars) and cytosolic (right group of bars) creatine kinase activity for the tibialis anterior extracts of the wild type (green bars, n=18 samples) and Δ*Mtln-1* (red bars, n=10 samples) male mice; (**g,h**) Mitochondrial (left group of bars) and cytosolic (right group of bars) creatine kinase activity for the soleus and tibialis anterior extracts of the wild type (green bars, n=3 samples) and Δ*Mtln-2* (red bars, n=3 samples) male mice; For all panels interquartile ranges are shown as solid bars, while all data range by thin lines. Horizontal line corresponds to median, while cross to the average. Significance level calculated accordingly to the Student's t-test is shown.

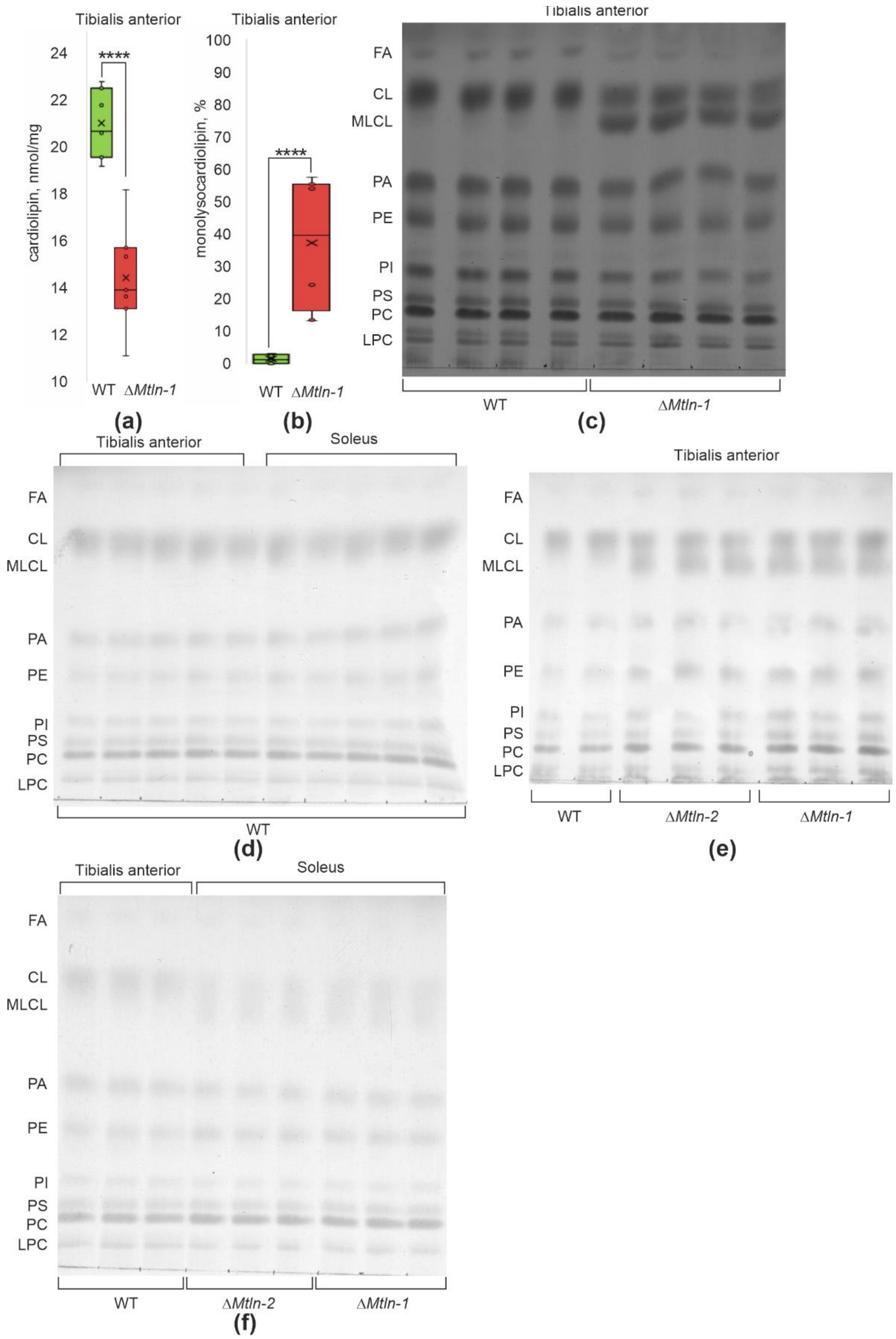

Tibialis anterior
cardiolipin, nmol/mg
24
22
20
18
16
14
12
10
****
WT ΔMtln-1
(a)
Tibialis anterior
monolysocardiolipin, %
100
90
80
70
60
50
40
30
20
10
0
****
WT ΔMtln-1
(b)
Tibialis anterior
FA
CL
MLCL
PA
PE
PI
PS
PC
LPC
WT
ΔMtln-1
(c)
Tibialis anterior
Soleus
FA
CL
MLCL
PA
PE
PI
PS
PC
LPC
WT
(d)
Tibialis anterior
FA
CL
MLCL
PA
PE
PI
PS
PC
LPC
WT
ΔMtln-2
ΔMtln-1
(e)
Tibialis anterior
Soleus
FA
CL
MLCL
PA
PE
PI
PS
PC
LPC
WT
ΔMtln-2
ΔMtln-1
(f)

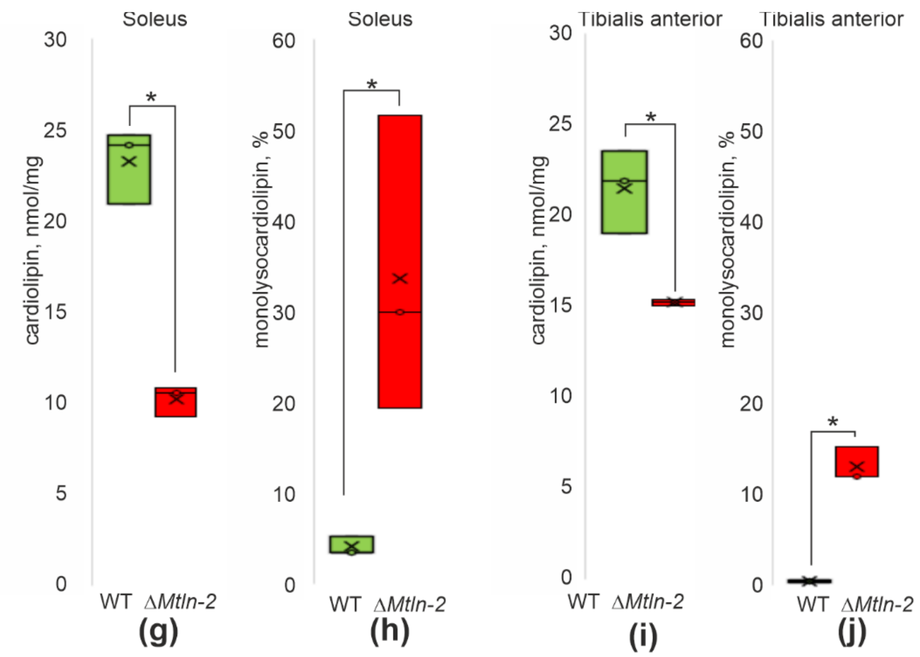

Supplementary Figure S6. Influence of *Mtln* gene inactivation on cardiolipin amount and integrity (related to the main text Figure 4). (**a**) Cardiolipin quantitation in the tibialis anterior mitochondria of the wild type (green bars, n=6) and Δ*Mtln-1* (red bars, n=7) mice; (**b**) Quantitation of the amount of monolysocardiolipin (MLCL) relative to the total amount of cardiolipin and monolysocardiolipin (MLCL+CL) in the tibialis anterior mitochondria of the wild type (green bars, n=7) and Δ*Mtln-1* (red bars, n=8) mice; (**c-f**) Primary data on thin layer chromatography of muscle mitochondrial lipids of the wild type male (n=5), Δ*Mtln-1* male (n=7) and Δ*Mtln-1* male (n=3) mice. Lipid designations are: fatty acids (FA), cardiolipin (CL), monolysocardiolipin (MLCL), phosphatidic acid (PA), phosphatidylethanolamine (PE), phosphatidylinositol (PI), phosphatidylserine (PS), phosphatidylcholine (PC), lysophosphatidylcholine (LPC); (**g,h**) Cardiolipin quantitation in the soleus and tibialis anterior mitochondria of the wild type (green bars, n=3) and Δ*Mtln-2* (red bars, n=3) mice; (**i,j**) Quantitation of the amount of monolysocardiolipin (MLCL) relative to the total amount of cardiolipin and monolysocardiolipin (MLCL+CL) in the soleus and tibialis anterior mitochondria of the wild type (green bars, n=3) and Δ*Mtln-2* (red bars, n=3) mice; For panels a,b,d-g interquartile ranges are shown as solid bars, while all data range by thin lines. Horizontal line corresponds to median, while cross to the average. Significance level calculated accordingly to the Student's t-test is shown.

Supplementary Table S2. Gene set enrichment analysis for the upregulated genes[1] in the soleus muscle upon *Mtln* gene inactivation (related to the main text Figure 1).

GO term molecular function	Set size	Enrichment score	p-value	FDR q-value	Familywise error rate p-value
MHC_PROTEIN_COMPLEX_BINDING	23	0.77	0	0.025	0.021
IMMUNE_RECEPTOR_ACTIVITY	136	0.56	0	0.031	0.05
MHC_CLASS_I_PROTEIN_BINDING	36	0.68	0.002	0.04	0.093
MANNOSE_BINDING	30	0.67	0	0.036	0.112
OPSONIN_BINDING	21	0.72	0	0.039	0.152
CHEMOKINE_ACTIVITY	41	0.63	0	0.034	0.157
ARACHIDONIC_ACID_MONOOXYGENASE_ACTIVITY	51	0.61	0	0.031	0.165
HYDROLASE_ACTIVITY_ACTING_ON_GLYCOSYL_BONDS	140	0.52	0	0.032	0.192
FERROUS_IRON_BINDING	35	0.64	0	0.03	0.205
COMPLEMENT_BINDING	27	0.67	0	0.029	0.219
MONOCARBOXYLIC_ACID_BINDING	93	0.54	0	0.027	0.223
OXIDOREDUCTASE_ACTIVITY_ACTING_ON_CH_OR_CH2_GROUPS	27	0.66	0	0.031	0.273
SERINE_TYPE_ENDOPEPTIDASE_INHIBITOR_ACTIVITY	130	0.52	0	0.032	0.292
HYDROLASE_ACTIVITY_HYDROLYZING_O_GLYCOSYL_COMPOUNDS	99	0.54	0	0.029	0.292
CARBON_OXYGEN_LYASE_ACTIVITY	82	0.55	0	0.033	0.333

LONG_CHAIN_FATTY_ACID_OMEGA_1_HYDROXYLASE_ACTIVITY	15	0.76	0	0.031	0.335
OLIGOSACCHARIDE_BINDING	18	0.72	0.004	0.03	0.345
IMMUNOGLOBULIN_BINDING	25	0.66	0.002	0.032	0.379
ANTIOXIDANT_ACTIVITY	86	0.54	0	0.035	0.426
HYDRO_LYASE_ACTIVITY	64	0.57	0.002	0.037	0.463
MHC_PROTEIN_COMPLEX_BINDING	23	0.77	0	0.025	0.021
IMMUNE_RECEPTOR_ACTIVITY	136	0.56	0	0.031	0.05
MHC_CLASS_I_PROTEIN_BINDING	36	0.68	0.002	0.04	0.093
MANNOSE_BINDING	30	0.67	0	0.036	0.112

[1] Transcriptome of Δ*Mtln-1* male mice (n=3) were compared with that of the wild type male mice (n=3) with GSEA method [15].

Supplementary Table S3. Gene set enrichment analysis for the downregulated genes[1] in the soleus muscle upon *Mtln* gene inactivation (related to the main text Figure 1).

GO term molecular function	Set size	Enrichment score	p-value	FDR q-value	Familywise error rate p-value
STRUCTURAL_CONSTITUENT_OF_RIBOSOME	163	-0.61	0	0	0
MECHANOSENSITIVE_ION_CHANNEL_ACTIVITY	17	-0.73	0.002	0.225	0.422
PROLACTIN_RECEPTOR_BINDING	28	-0.64	0	0.249	0.596
HISTONE_METHYLTRANSFERASE_ACTIVITY_H3_K4_SPECIFIC	18	-0.7	0.002	0.197	0.621
EPHRIN_RECEPTOR_BINDING	32	-0.62	0	0.18	0.672
MOLECULAR_FUNCTION_INHIBITOR_ACTIVITY	60	-0.55	0	0.156	0.685
NUCLEOBASE_CONTAINING_COMPOUND_TRANSMEMBRANE_TRANSPORTER_ACTIVITY	50	-0.56	0.004	0.16	0.746
ARP2_3_COMPLEX_BINDING	17	-0.69	0.01	0.157	0.784
HYALURONIC_ACID_BINDING	26	-0.62	0.01	0.185	0.861
TRANSCRIPTION_REGULATOR_INHIBITOR_ACTIVITY	19	-0.67	0.008	0.169	0.862
OXIDOREDUCTASE_ACTIVITY_ACTING_ON_A_HEME_GROUP_OF_DONORS	19	-0.67	0.013	0.23	0.954
VASCULAR_ENDOTHELIAL_GROWTH_FACTOR_RECEPTOR_BINDING	16	-0.67	0.016	0.25	0.976
PHEROMONE_RECEPTOR_ACTIVITY	100	-0.48	0	0.232	0.977
RECEPTOR_SERINE_THREONINE_KINASE_BINDING	34	-0.58	0.02	0.237	0.987
FIBROBLAST_GROWTH_FACTOR_RECEPTOR_BINDING	29	-0.59	0.002	0.222	0.987
TRANSMEMBRANE_RECEPTOR_PROTEIN_TYROSINE_KINASE_ACTIVITY	61	-0.51	0.01	0.215	0.988
SNRNA_BINDING	43	-0.54	0.002	0.215	0.99
EXTRACELLULAR_MATRIX_STRUCTURAL_CONSTITUENT_CONFERRING_TENSILE_STRENGTH	38	-0.54	0.006	0.24	0.997
INSULIN_RECEPTOR_BINDING	28	-0.57	0.02	0.233	0.997
DOPAMINE_RECEPTOR_BINDING	25	-0.59	0.02	0.239	0.999

[1] Transcriptome of Δ*Mtln-1* male mice (n=3) were compared with that of the wild type male mice (n=3) with GSEA method [15].

Supplementary References

1. Averina, O.A.; Permyakov, Oleg A, O.A.; Mariia A. Emelianova; Olga O. Grigoryeva; Mikhail V. Gulyaev; Olga S. Pavlova; Sofia S. Mariasina; Olga Yu. Frolova; Marina V. Kurkina; Galina V. Baydakova; et al. Mitochondrial Peptide Mtln Contributes to Oxidative Metabolism in Mice. *Biochimie*.
2. Rogers, D.C.; Peters, J.; Martin, J.E.; Ball, S.; Nicholson, S.J.; Witherden, A.S.; Hafezparast, M.; Latcham, J.; Robinson, T.L.; Quilter, C.A.; et al. SHIRPA, a Protocol for Behavioral Assessment: Validation for Longitudinal Study of Neurological Dysfunction in Mice. *Neurosci. Lett.* **2001**, *306*, 89–92, doi:10.1016/s0304-3940(01)01885-7.

3. Wang, C.; Yue, F.; Kuang, S. Muscle Histology Characterization Using H&E Staining and Muscle Fiber Type Classification Using Immunofluorescence Staining. *Bio Protoc* **2017**, *7*, doi:10.21769/BioProtoc.2279.
4. Pasut, A.; Jones, A.E.; Rudnicki, M.A. Isolation and Culture of Individual Myofibers and Their Satellite Cells from Adult Skeletal Muscle. *J Vis Exp* **2013**, e50074, doi:10.3791/50074.
5. *Pathology of the Mouse: Reference and Atlas*; Maronpot, R.R., Ed.; 1. ed.; Cache River Press: Saint Louis, Mo, 1999; ISBN 978-1-889899-02-2.
6. Hard, C. C.; Alden, C. L.; Bruner, R. H. G.; Frith, C. H.; Lewis, R. M.; Owen, R. A.; Krieg, K.; Durchfeld-Meyer, B. Non-Proliferative Lesions of the Kidney and Lower Urinary Tract in the Rat. In *URG-1, Guides for Toxicological Pathology*; STP/ARP/AFIP: Washington, D.C., 1999.
7. *Bancroft's Theory and Practice of Histological Techniques*; Suvarna, S.K., Layton, C., Bancroft, J.D., Eds.; Eighth edition.; Elsevier: Amsterdam, 2019; ISBN 978-0-7020-6864-5.
8. Vyssokikh, M.Y.; Holtze, S.; Averina, O.A.; Lyamzaev, K.G.; Panteleeva, A.A.; Marey, M.V.; Zinovkin, R.A.; Severin, F.F.; Skulachev, M.V.; Fasel, N.; et al. Mild Depolarization of the Inner Mitochondrial Membrane Is a Crucial Component of an Anti-Aging Program. *Proc Natl Acad Sci U S A* **2020**, *117*, 6491–6501, doi:10.1073/pnas.1916414117.
9. Gnaiger, E.; Kuznetsov, A.V.; Schneeberger, S.; Seiler, Rü.; Brandacher, G.; Steurer, W.; Margreiter, R. Mitochondria in the Cold. In *Life in the Cold*; Heldmaier, G., Klingenspor, M., Eds.; Springer Berlin Heidelberg: Berlin, Heidelberg, 2000; pp. 431–442 ISBN 978-3-642-08682-3.
10. Bucher, T.; Luh, E.; Pette, D. Einfache Und Zusammengesetzte Optische Tests Mit Pyridininucleotiden. In *Handbuch der physiologisch- und pathologisch-chemischen Analyse*; Hoppe-Seyler/Thierfelder; Lang, K. and Lehnartz, E.: Berlin, 1964; Vol. VI/A, pp. 292–339.
11. Wallimann, T.; Tokarska-Schlattner, M.; Schlattner, U. The Creatine Kinase System and Pleiotropic Effects of Creatine. *Amino Acids* **2011**, *40*, 1271–1296, doi:10.1007/s00726-011-0877-3.
12. Bligh, E.G.; Dyer, W.J. A Rapid Method of Total Lipid Extraction and Purification. *Can J Biochem Physiol* **1959**, *37*, 911–917, doi:10.1139/o59-099.
13. Pinault, M.; Guimaraes, C.; Dumas, J.; Servais, S.; Chevalier, S.; Besson, P.; Goupille, C. A 1D High Performance Thin Layer Chromatography Method Validated to Quantify Phospholipids Including Cardiolipin and Monolysocardiolipin from Biological Samples. *Eur. J. Lipid Sci. Technol.* **2020**, *122*, 1900240, doi:10.1002/ejlt.201900240.
14. Dobin, A.; Davis, C.A.; Schlesinger, F.; Drenkow, J.; Zaleski, C.; Jha, S.; Batut, P.; Chaisson, M.; Gingeras, T.R. STAR: Ultrafast Universal RNA-Seq Aligner. *Bioinformatics* **2013**, *29*, 15–21, doi:10.1093/bioinformatics/bts635.
15. Subramanian, A.; Tamayo, P.; Mootha, V.K.; Mukherjee, S.; Ebert, B.L.; Gillette, M.A.; Paulovich, A.; Pomeroy, S.L.; Golub, T.R.; Lander, E.S.; et al. Gene Set Enrichment Analysis: A Knowledge-Based Approach for Interpreting Genome-Wide Expression Profiles. *Proc. Natl. Acad. Sci. U.S.A.* **2005**, *102*, 15545–15550, doi:10.1073/pnas.0506580102.
16. Ashburner, M.; Ball, C.A.; Blake, J.A.; Botstein, D.; Butler, H.; Cherry, J.M.; Davis, A.P.; Dolinski, K.; Dwight, S.S.; Eppig, J.T.; et al. Gene Ontology: Tool for the Unification of Biology. The Gene Ontology Consortium. *Nat Genet* **2000**, *25*, 25–29, doi:10.1038/75556.
